# Therapeutics Targeting the Core Apoptotic Machinery

**DOI:** 10.3390/cancers13112618

**Published:** 2021-05-26

**Authors:** Claudia Hamilton, Jennifer P. Fox, Daniel B. Longley, Catherine A. Higgins

**Affiliations:** Patrick G. Johnston Centre for Cancer Research, Queens University, Belfast BT9 7BL, UK; chamilton40@qub.ac.uk (C.H.); Jfox14@qub.ac.uk (J.P.F.); d.longley@qub.ac.uk (D.B.L.)

**Keywords:** apoptosis, cancer therapeutics, resistance, FLIP

## Abstract

**Simple Summary:**

Cancer develops when the balance between cell death and cell division in tissues is dysregulated. A key focus of cancer drug discovery is identifying therapeutic agents which will selectively kill and eliminate cancer cells from the body. A number of proteins can prevent the death of cancer cells and developing inhibitors against these proteins to promote cancer cell death is a focus of recent drug discovery efforts. This review aims to summarize the key targets being explored, the drug development approaches being adopted, and the success or limitations of agents currently approved or in clinical development.

**Abstract:**

Therapeutic targeting of the apoptotic pathways for the treatment of cancer is emerging as a valid and exciting approach in anti-cancer therapeutics. Accumulating evidence demonstrates that cancer cells are typically “addicted” to a small number of anti-apoptotic proteins for their survival, and direct targeting of these proteins could provide valuable approaches for directly killing cancer cells. Several approaches and agents are in clinical development targeting either the intrinsic mitochondrial apoptotic pathway or the extrinsic death receptor mediated pathways. In this review, we discuss the main apoptosis pathways and the key molecular targets which are the subject of several drug development approaches, the clinical development of these agents and the emerging resistance factors and combinatorial treatment approaches for this class of agents with existing and emerging novel targeted anti-cancer therapeutics.

## 1. Introduction

**Figure 1:**
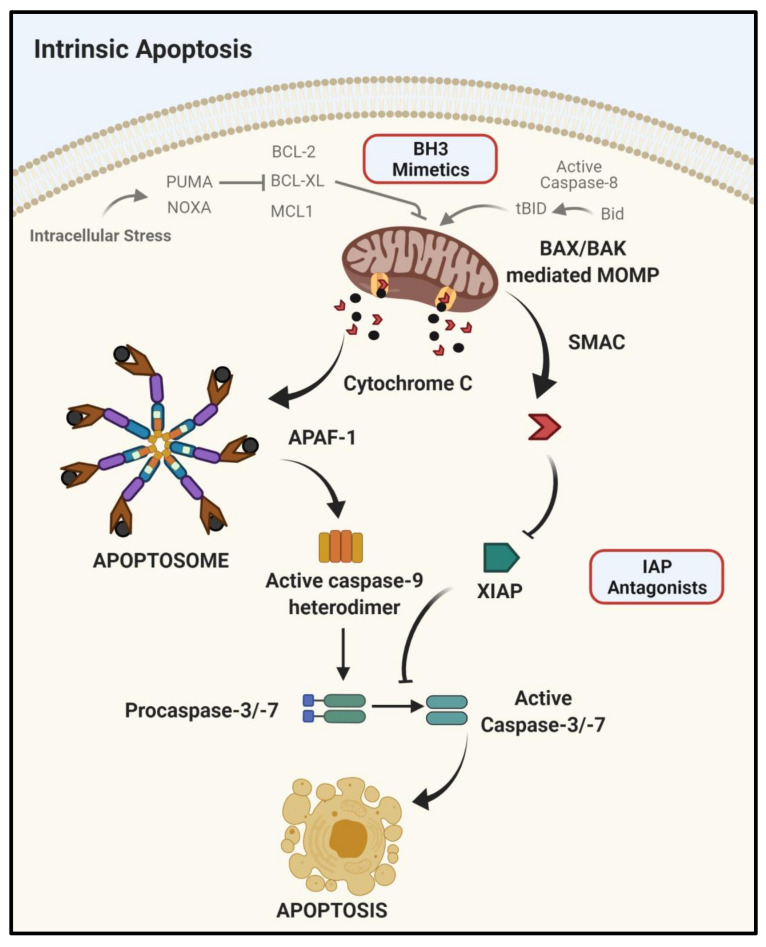
Intrinsic apoptotic pathway. In the intrinsic pathway, internal stresses such as DNA damage can lead to the B-cell lymphoma family-2 homology domain -3 (BH3) sensor proteins being activated and inhibiting anti-apoptotic B-cell lymphoma family-2 (BCL-2) proteins, leading to oligomerisation and activation of BCL-2 associated X protein (BAX)and BCL-2 antagonist killer 1 (BAK) and the formation of pores in the outer mitochondrial membrane. Mitochondrial outer membrane permeabilization (MOMP) releases cytochrome c and second mitochondrial activator of caspase (SMAC). Cytochrome c forms a complex with apoptotic protease-activating factor 1 (APAF1) and pro-caspase-9, termed the apoptosome, in which procaspase-9 dimerises and becomes activated triggering the activation of a caspase cascade. The active caspase-9 heterodimer cleaves and activates the apoptotic effector caspases -3 and -7. SMAC inhibits X-linked inhibitor of apoptosis (XIAP) to facilitate activation of procaspase-3 and -7. Created with BioRender.com (accessed on 25 May 2021).

**Figure 2:**
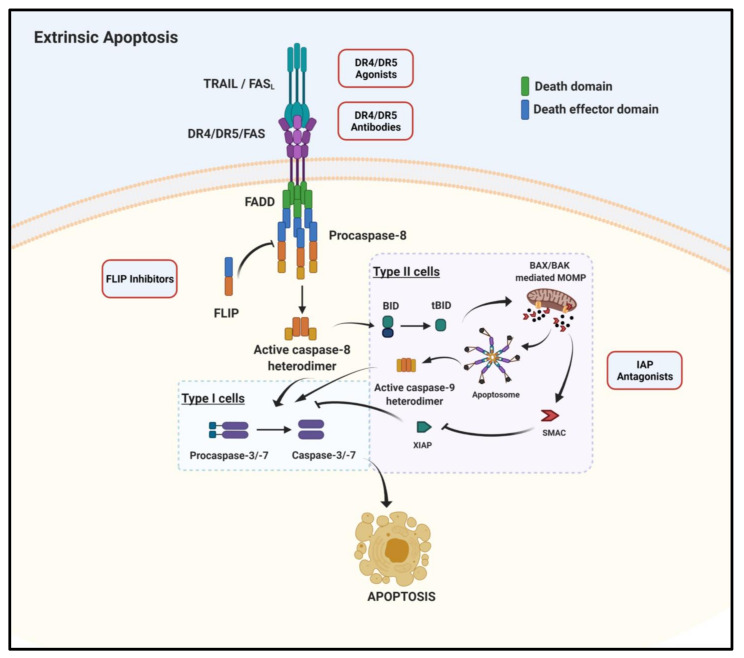
The extrinsic apoptosis pathway. The death receptor-mediated extrinsic apoptosis pathway is initiated following ligand binding to a trimeric death receptor. In the case of Tumor necrosis factor (TNF)-related apoptosis-inducing ligand (TRAIL) binding the TRAIL receptor (TRAIL-R) the adaptor protein, Fas-associated death domain (FADD), is recruited to the death receptor via homotypic death domain interactions. FADD subsequently recruits procaspase-8 through interactions of their respective death effector domains, forming the death inducing signaling complex (DISC). Procaspase-8 dimerizes and is activated to release the active caspase-8 homodimer. FADD-like IL1β-converting enzyme inhibitory protein (FLIP) can also be recruited to the DISC where it can modulate the activation of caspase-8. In Type I cells, caspase-8 directly activates effector procaspase-3 and -7. In type II cells, apoptosis depends on caspase-8-mediated cleavage of BH3-interacting domain death agonist (BID). Activated tBID translocates to the mitochondria to induce BCL-2 associated X protein (BAX)/ BCL-2 antagonist killer 1 (BAK) mediated mitochondrial outer membrane permeabilization (MOMP). Subsequent second mitochondrial activator of caspase (SMAC) release neutralizes X-linked inhibitor of apoptosis (XIAP) and permits the final auto-catalytic step in the activation of caspases-3/7. Created with BioRender.com (accessed on 25 May 2021).

A key focus of cancer drug discovery is identifying therapeutic agents which will selectively kill cancer cells. Apoptosis is a physiological, programmed form of cell death that plays a central role in maintaining tissue homeostasis and eliminating damaged or infected cells. Resistance to apoptotic cell death is a recognized hallmark of cancer, making the molecular drivers of the anti-apoptotic response appealing anti-cancer therapeutic targets [1,2]. Cancer cells are dependent on a relatively small number of anti-apoptotic proteins for their survival, including the anti-apoptotic B-cell lymphoma family (BCL-2) proteins, inhibitors of apoptosis proteins (IAPs) and cellular Fas-associated death domain (FADD)-like IL1β (Interleukin-1β)-converting enzyme-inhibitory protein (FLIP), all of which are established anti-cancer therapeutic targets [3]. Two distinct pathways of apoptotic cell death have been described: the intrinsic mitochondrial-mediated pathway (Figure 1) and the extrinsic death receptor (DR)-mediated pathway (Figure 2). Both pathways are well described and tightly regulated by a family of cysteine proteases known as the caspases [4]. Caspases (cysteinyl aspartate-specific proteases) are synthesized as inactive zymogens and activated in a hierarchical manner by homodimerization or cleavage by other caspases. Both pathways ultimately result in the activation of initiator caspases (caspases 8, 9 and 10) which activate downstream effector caspases (caspases 3, 6 and 7), triggering a caspase cascade leading to the characteristic biochemical and morphological changes associated with apoptosis [5].

### 1.1. Intrinsic Apoptosis

Intrinsic apoptosis is initiated internally within the cell in response to various stimuli including DNA damage, reactive oxygen species or a lack of essential survival signaling. Intrinsic mitochondrial mediated apoptosis is tightly controlled by the BCL-2 family of pro- and anti-apoptotic proteins that balance the decision between life and death [6]. BCL-2 family members can be grouped into three distinct classes based on their structure and function: the anti-apoptotic members containing four tandem BCL-2 homology (BH1–4) domains that promote cell survival; the pro-apoptotic BH3-only proteins with a single BH domain that promote cell death and the pro-apoptotic effector proteins BCL-2-associated X protein (BAX) and BCL-2 antagonist killer 1 (BAK). BCL-2 family members reside on the outer mitochondrial membrane and function through direct protein-protein interactions with other family members, involving their BH3 domains. Pro-apoptotic BAX and BAK oligomerize forming pores in the mitochondrial outer membrane leading to mitochondrial outer membrane permeabilization (MOMP) that allows the release of cytochrome c, second mitochondria-derived activator of caspases (SMAC) and other pro-apoptotic proteins into the cytoplasm. Cytochrome c forms a complex with apoptotic protease-activating factor 1 (APAF1) and procaspase-9, termed the apoptosome, in which procaspase-9 dimerizes and becomes activated triggering the activation of a caspase cascade. Anti-apoptotic BCL-2 proteins, such as BCL-2, B-cell lymphoma-extra-large (BCL-X_L_) and Myeloid cell leukemia sequence 1 (MCL-1) can bind to and inhibit BAX and BAK oligomerization. BH3 proteins like Bcl-2-like protein 11 (BIM), p53 upregulated modulator of apoptosis (PUMA), Phorbol-12-myristate-13-acetate-induced protein 1 (NOXA) and BH3 interacting-domain death agonist (BID) are activated in response to specific cellular stresses/signals and promote the oligomerization of BAX and BAK either through direct activation or by binding to BCL-2/BCL-X_L_/MCL-1, thereby blocking their inhibition of BAX/BAK oligomerization leading to MOMP. Overall, the BCL-2 family members act as cellular sensors which determine the fate of the cell in response to specific stresses and whether the balance will tip in favor of apoptosis [3,7,8,9].

### 1.2. Extrinsic Apoptosis

The DR mediated extrinsic apoptotic pathway is activated following ligand binding, such as binding of tumor necrosis factor (TNF)-related apoptosis-inducing ligand (TRAIL) to TRAIL-R1 (DR4)/TRAIL-R2 (DR5) at the plasma membrane leading to the formation of a death-inducing signaling complex (DISC). The DISC multiprotein complex comprises the death receptor, the adaptor protein Fas-Associated Death Domain (FADD) and procaspase-8, which can recruit additional cell death regulators, most notably the pseudo-caspase and procaspase-8 paralog, FLIP [10,11].

DISC proteins interact through well characterized death domains (DD) and death effector domains (DED) [10,12]. The intracellular DDs of DRs engage with FADD through homotypic DD interactions exposing the FADD N-terminal DED. The adaptor protein can then engage in homotypic interactions with other DED containing proteins such as the tandem DED containing proteins procaspase-8 and FLIP. FLIP is a key regulator of the extrinsic cell death pathway controlling the initiation and extent of caspase-8 activation at the DISC. Proximity-induced homodimerization of procaspase-8 results in conformational changes in its catalytic domain that leads to its activation and the initiation of the proteolytic apoptotic cascade. Active caspase-8 can subsequently cleave the executioner procaspases-3 and -7 in the first step of their activation and the BH3-protein BID, which when truncated (tBID) by caspase-8 translocates to the mitochondria to promote MOMP. FLIP is capable of regulating caspase activation at the DISC with FLIP(L), the long splice form, capable of acting as an inhibitor or promoter of caspase-8 activation depending on the context and stoichiometry of the DISC proteins. FLIP(S), the short splice form, can heterodimerize with procaspase-8 preventing its processing and activation [12,13]. In “Type 1” cells, caspase-8 dependent processing of the executioner caspases is sufficient to induce apoptotic cell death independently of mitochondrial driven events. In “Type II” cells, the X-linked Inhibitor of apoptosis protein (XIAP) inhibits the second step in the activation of executioner caspases-3/7 (auto-catalytic activation) and, in these cells, further mitochondrial amplification via tBID is necessary. As a result of MOMP, SMAC and human serine protease high temperature requirement A (HTRA2) (HtrA2/Omi) are released in addition to cytochrome c; all of these pro-apoptotic proteins are able to block the anti-apoptotic activity of XIAP thereby amplifying the apoptotic cascade [4,14,15].

Resistance to apoptosis is not only a hallmark of cancer, but also a key cause of resistance to cancer therapy, with mechanisms that resist apoptosis being selected for during treatment. However, accumulating evidence suggests that rather than losing the effectors of apoptosis, many cancer cells enhance expression of a relatively small number of anti-apoptotic proteins and become “addicted” to these proteins for their survival [16]; therefore, direct targeting of these proteins provides valuable avenues for selectively eliminating cancer cells.

## 2. Therapeutic Strategies for Promoting Apoptosis Directly—Intrinsic Pathway

**Figure 3:**
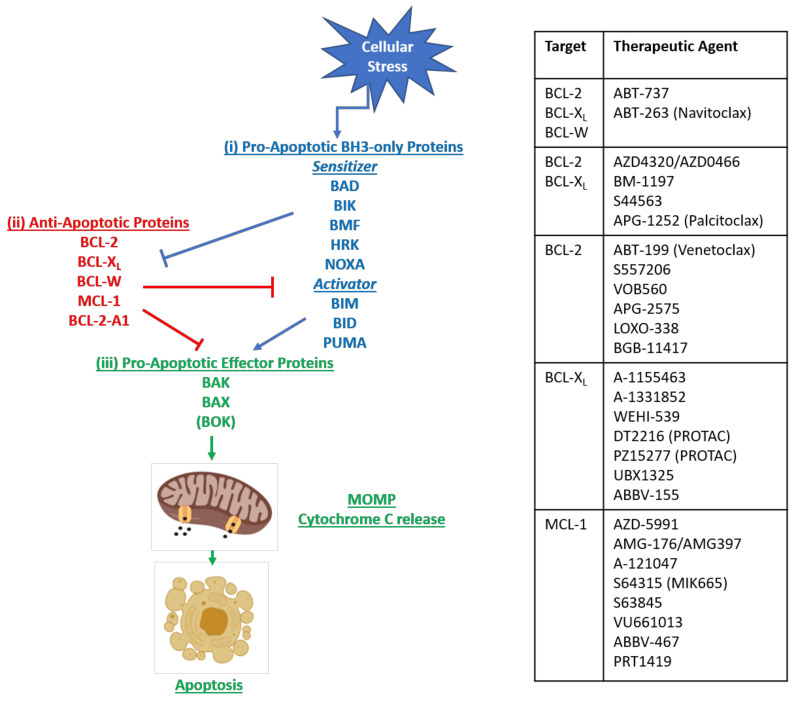
B-cell lymphoma family-2 (BCL-2) Family Members. Family members of the BCL-2 protein family comprising: (**i**) B-cell lymphoma family-2 homology domain -3 (BH3)-only pro-apoptotic initiating proteins; (**ii**) the anti-apoptotic family members that protect against apoptosis by inhibiting the BH3-only proteins or neutralising the effector proteins directly; and (**iii**) the pro-apoptotic effector proteins which once activated trigger mitochondrial outer membrane permeabilization (MOMP), releasing apoptogenic factors from the mitochondria, leading to caspase activation and apoptosis. Listed are the agents developed to target specifically the indicated target(s).

Over the past decade, new cancer treatments that directly promote apoptosis have emerged with drugs targeting the BCL-2 family of proteins the most clinically advanced. Members of the BCL-2 family play a central role in regulating cell death through pro- and anti-apoptotic intracellular signals [17]. The BCL-2 gene family encodes more than 20 proteins, and pharmacologically inhibiting the protein-protein interactions between pro- and anti-apoptotic members to promote MOMP and cell death was an exciting development in this field (Figure 3) [17].

Early attempts at targeting Bcl-2 led to the emergence of several agents which showed promise in the pre-clinical setting with a number advancing to clinical trials including Oblimersen (Genasense^®^, Genta Incorporated, La Jolla, California, USA), an antisense oligodeoxynucleotide designed to target Bcl-2 mRNA [18], the natural product gossypol which directly interacts with BCL-X_L_ displacing BH3 proteins and Obatoclax (Teva Pharmaceuticals (Gemin X), Parsippany, NJ, USA), a pan-Bcl-2 inhibitor [17,19,20]. Early agents demonstrated significant clinical toxicities with moderate efficacies halting their clinical development. Structure-based design determined by nuclear magnetic resonance (NMR) and fragment based-screening approaches enabled the development of more selective agents targeting specific BCL-2 family members [17,21]. These BH3 mimetics were non-peptide small molecule inhibitors that demonstrated potent and specific disruption of the interactions between the pro-and anti-apoptotic BCL-2 proteins typically binding into the hydrophobic grooves of anti-apoptotic proteins, neutralizing them. ABT-737, the first-in-class BH3 mimetic, binds with high affinity to BCL-2, BCL-X_L_ and B-cell lymphoma-w (BCL-W) subsequently disrupting the interactions with pro-apoptotic BH3-only proteins, demonstrated preclinical activity in a range of hematological and solid cancers but had poor pharmacokinetic (PK) properties [22,23,24]. An analogue of ABT-737, Navitoclax (ABT-263) (AbbVie, North Chicago, IL, USA), subsequently emerged as an orally available drug with enhanced PK properties (half-life of 8.9 h) with a Ki of < 1 nM against BCL-2, BCL-X_L_ and BCL-W and a lower affinity against MCL-1 (Ki 550 nM) [25]. Unfortunately, during its clinical development, thrombocytopenia emerged as a dose-limiting toxicity attributable to BCL-X_L_ inhibition [26,27]. Despite the setbacks positive acceptable toxicity, PK and efficacy data was reported for navitoclax in combination with rituximab in relapsed/refractory chronic lymphocytic leukemia patients (CLL) [28,29]. In addition, a number of clinical trials are ongoing with navitoclax in myelofibrosis (NCT04472598, NCT03222609, NCT04041050) and in combination with targeted agents in *KRAS* mutant solid cancers (NCT02079740) and *BRAF* mutant melanoma and other solid tumor settings (NCT01989585).

### 2.1. Selective BCL-2 Inhibitors

Selective Bcl-2 inhibitors were developed with the aim of overcoming the dose-limiting thrombocytopenia observed with pan- and dual-BCL-2 family inhibitors. Venetoclax (ABT-199) (AbbVie, North Chicago, IL, USA), was the first selective orally bioavailable Bcl-2 inhibitor to be developed with high affinity to BCL-2 but much lower affinity for BCL-X_L_ and BCL-W and no affinity towards MCL-1 [30,31]. Venetoclax induced apoptosis in hematological cancer cells dependent on Bcl-2 for their survival and tumor regression in preclinical xenograft models [26,32]. Early clinical studies with venetoclax focused on hematological malignancies, such as CLL, acute myeloid leukemia (AML) and multiple myeloma (MM) where significant improvements in overall objective responses were observed. Early trials however were halted due to the emergence of a rapid onset of tumor lysis syndrome where rapidly dying cancer cells release their contents into the blood often having fatal consequences. Altering the scheduling of venetoclax, using a ramp up approach, combined with careful clinical monitoring of patients, resulted in durable clinical responses, with venetoclax subsequently gaining FDA breakthrough therapy designation in 2015 and approval for the treatment of CLL in 2016 for del(17p) CLL patients [33,34,35]. In addition, venetoclax received accelerated approval for use in combination with either azacytidine, decitabine or cytarabine for AML patients ineligible for intensive induction chemotherapy [36]. Venetoclax has undoubtedly demonstrated the effectiveness of this class of therapeutics with its approval and clinical use in CLL and AML patients and a broad number of advanced trials ongoing across other hematological settings including lymphoma, MM and myelodysplastic syndromes. In addition, clinical trials are also evaluating this class of agent in a number of solid cancer settings such as in combination with pembrolizumab in non-small cell lung cancer (NSCLC) (NCT04274907) and with palbociclib (Pfizer, NY, USA) and letrozole (Novartis, Cambridge, MA, USA) in estrogen receptor and Bcl-2 positive breast cancers (NCT03900884), Given the success of venetoclax others are not surprisingly actively pursuing this area. Additional small molecules, BGB-11417 (Beigene, Beijing, China) and LOXO-338 (Loxo Oncology, Stamford, CT, USA), are emerging with reported improvement in PK properties and improved efficacy compared to venetoclax in AML and a range of lymphoma xenograft models [37,38] with BGB-11417 recently progressing to clinical trials for hematological malignancies (NCT04277637 and NCT04771130).

### 2.2. Dual BCL-2/BCL-X_L_

Several other BH3 mimetics are emerging and progressing preclinically, including BM-1197, S44563 and BCL2–32 which bind to BCL-2 and BCL-X_L_ with nanomolar affinity [33,39,40,41]. Dual BCL-2/BCL-X_L_ inhibitors are anticipated to deliver therapeutic benefit in many hematological and solid cancers, but their clinical development has also been limited by tolerability and safety issues including thrombocytopenia. The dual BCL-2/BCL-X_L_ compound AZD4320 (AstraZeneca, Cambridge, UK) progressed to clinical development with IV administration and intermittent scheduling approaches to enable recovery from the on-target side effects; however, cardiovascular toxicity was observed during preclinical development, halting its progress. To overcome challenging PK properties and dose-limiting toxicities, a nanocarrier delivery approach for AZD4320 was developed conjugating it to the clinically validated DEP® dendrimer platform AZD0466 (Starpharma, Preston, Victoria, Australia) [42]. Delivering this compound via a drug-dendrimer conjugate improved its therapeutic index enabling its progression into a phase 1 clinical trial (NCT04214093). Palcitoclax (APG-1252) (Ascentage Pharma, Suzhou, China) has also been evaluated in the clinical setting where it demonstrated tolerability and a favorable toxicity profile supporting its further development in SCLC and other solid tumors (NCT03080311, NCT03387332) [43,44].

### 2.3. Selective BCL-X_L_ Inhibitors

The BCL-2 family member BCL-X_L_ is known to have an anti-apoptotic role in several solid cancers and some hematological malignancies making it an additional target for cancer therapeutics. The neutropenia observed with Navitoclax, in combination with chemotherapy, limited its clinical use and was hypothesized to be attributable to BCL-2 inhibition in the solid tumor setting exacerbating the neutropenic toxicity of these chemotherapies [45,46]. As a result, selective inhibitors of BCL-X_L_ were hypothesized to maintain efficacy in the solid tumor setting, in combination with chemotherapy, while avoiding the dose-limiting neutropenia observed with the dual targeting agents. Several BCL-X_L_ inhibitors have been reported, including WEHI-539, A-1155463, A-1331852 and DT2216 [45,47,48]. Thrombocytopenia was the predominant clinical dose limiting toxicity observed with dual targeting agents and attributable to BCL-X_L_ inhibition. Recently described proteolysis targeting chimeras (PROTACs), offer the potential to mitigate the on-target thrombocytopenia traditionally associated with BCL-X_L_ inhibition. DT2216, a first-in-class selective BCL-X_L_ degrader, is reported to target tumor cells more selectively as it targets BCL-X_L_ to the Von Hippel-Lindau (VHL) E3 ligase for proteasomal degradation; platelets have minimal expression of VHL [47,49]. DT2216 has demonstrated preclinical activity against BCL-X_L_ -dependent T cell lymphomas without causing significant platelet toxicity specifically degrading BCL-X_L_ while sparing BCL-2 [47,49]. An additional similar PROTAC, PZ15227, has also recently emerged which induces BCL-X_L_ polyubiquitination and degradation [50]. BCL-X_L_ has been reported to be highly expressed in the tumor-infiltrating regulatory T-cells (Treg) population in several cancers. Both PROTAC compounds have demonstrated induction of apoptosis in Tregs and the activation of tumor infiltrating CD8+ T cells resulting in a decrease in tumor growth in immunocompetent tumor models; this suggests that targeting of BCL-X_L_ may also have the potential to improve cancer immunotherapy [50]. UBX1325 (Unity Biotechnology, San Franscisco, CA, USA), another specific BCL- X_L_ inhibitor, is also being evaluated in patients with diabetic macular edema widening the scope of this class of agents beyond cancer (NCT04537884, NCT04857996). An alternative approach using a BCL-X_L_ targeting antibody drug conjugate (ABBV-155, AbbVie, North Chicago, IL, USA), is currently being evaluated in a phase I trial as monotherapy and in combination with docetaxel or paclitaxel for small cell lung cancer, NSCLC, and breast cancer (NCT03595059) [51,52].

### 2.4. Selective MCL1 Inhibitors

MCL-1, an anti-apoptotic protein of the BCL-2 family, is commonly overexpressed in cancers and plays a critical role in promoting cell survival. MCL-1 has also been implicated as a resistance mechanism to conventional chemo- and radio-therapies as well as other targeted agents including resistance to BH3 mimetic therapy making it an attractive therapeutic target [17]. Early MCL-1 inhibitors lacked specificity, however NMR-based screening, as with other BH3 mimetics, identified a large hydrophobic pocket in the P2 region of the protein that enabled more selective inhibitors to be designed [17]. Multiple approaches have identified small molecules with high affinity binding to MCL-1 and potent activity against MCL-1-dependent cells [17,53,54,55,56,57]. S63845, an early specific inhibitor of MCL-1, bound to its BH3 domain and inactivated the anti-apoptotic function of MCL-1 (K_D_ = 0.19 nM)[58,59]. S64315 (MIK665) was evaluated in a phase 1 study (NCT02992483) and has subsequently progressed to phase II studies in hematological malignancies in combination with VOB560, a novel Bcl-2 inhibitor ((Novartis, Cambridge, MA, USA) (NCT04702425) and azacytidine (NCT04629443) and venetoclax (NCT03672695) in acute myeloid leukemia (AML).

AMG-176 was the first MCL-1 specific inhibitor to enter clinical trial (NCT03797261) closely followed by AZD5991[60,61,62]. However, in November 2019 the U.S Food and Drug Administration (FDA) placed a clinical hold on a phase 1 dose escalation study of AMG-397, an oral MCL-1 inhibitor in clinical development, following Amgen’s findings of cardiotoxicity in patients on the trial. Subsequently, Amgen (Thousand Oaks, CA, USA) halted the clinical trial of AMG-176 [63]. MCL-1 had previously been shown to be important for normal tissue physiology, including cardiac homeostasis, with early studies demonstrating cardiomyocyte-specific *Mcl-1* knockout in mice resulting in the development of a rapid and fatal cardiomyopathy due to mitochondrial swelling and rupture [64]. Additional conditional knockout studies have shown that MCL-1 is essential for the development and survival of several additional cell types including hematopoietic stem cells, B and T-lymphocytes, granulocytes, macrophages, and neurons[65,66]. Despite this early setback an additional MCL-1 inhibitor, PRT1419 (Prelude Therapeutics, Wilmington, DE, USA), is progressing as an orally bioavailable agent in hematological malignancies (NCT04543305) and an additional iv formulation has been granted FDA approval as an investigational new drug (IND) application for evaluation in solid cancers including sarcoma, melanoma, lung, and breast cancer (NCT04837677). Further clinical evaluation of these agents will further assess their tolerability and efficacy in patients and how best to schedule them for optimal efficacy and safety.

### 2.5. Alternative Approaches

A novel approach to inhibiting Bcl-2 using a DNA antisense strategy was previously described using a Bcl-2 targeted liposomal formulation. The novel compound, PNT2258 (ProNai Therapeutics, Vancouver, BC, Canada), demonstrated downregulation of Bcl-2 at the promoter, mRNA, and protein levels ultimately resulting in apoptosis [67]. PNT2258 was investigated in Phase 1 and Phase 2 clinical trials in lymphoma patients [68]. The first-in-human study successfully delivered a native unmodified DNA oligonucleotide via a protective liposomal nanoparticle and identified a maximum tolerated dose of exposure; however, following the subsequent phase 2 studies in lymphoma patients, the development of PNT2258 was suspended due to lack of robust results to warrant its further clinical investigation.

BCL-2 proteins are typically characterized by the presence of 4 BH homology domains but exert their antiapoptotic role primarily through the BH1, BH2 and BH3 domains. The BH4 region is characterized by sequences of 18–20 amino acids in the N-terminal domain of BCL-2 and BCL-X_L_ and other antiapoptotic members of the family and is generally responsible for the interaction with other non-BH domain-containing proteins and critically involved in the regulation of non-canonical apoptotic cellular functions, including cell differentiation and proliferation [69]. The cleavage of the BH4 domain in BCL-X_L_ and BCL-2 by caspases results in the loss of the NH_2_-terminal BH4 homology domain that is required for their anti-apoptotic activity converting these antiapoptotic proteins into proapoptotic proteins that can induce cell death [70]. Targeting the BH4 domain diminishes the anti-apoptotic function of BCL-2 hence it is emerging as an alternative strategy for targeting Bcl-2 family members. Novel therapeutic approaches targeting the BH4 domain to target cancer growth and progression are thus emerging [71].

### 2.6. BCL-2 Family Members—Emerging Resistance and Combination Approaches

The success of targeting BCL-2 family members clinically, particularly with the FDA approval of venetoclax, has demonstrated the clinical potential of this class of agents and transformed the approaches to targeting hematological cancers. Emerging resistance mechanisms have been described with strategies to overcome these under clinical investigation (Table 1) [72,73,74]. A recent search (April 2021) on ClinicalTrials.gov indicates over 250 active trials in cancer with venetoclax both as monotherapy and in combination with standard-of-care and other novel targeted agents to tackle the emerging resistance [26]. Resistance mechanisms are often linked to other anti-apoptotic proteins, for example MCL-1 is an important mediator of resistance to ABT-737 and other chemotherapeutics [6]. Combinations with other agents including inhibition of alternative pathway targets such as inhibitor of apoptosis proteins (IAPs) are thus emerging as promising combinations. Conversely several combination partners for MCL-1 inhibitors are being explored in preclinical studies, including combinations with other BH3 mimetics and several standard-of-care treatment regimes, however given recent safety concerns, the toxicity issues need to be fully understood to determine if a safe therapeutic window can exist for combining these agents.

## 3. Inhibitor of Apoptosis Proteins (IAP)

IAPs are frequently overexpressed in a variety of cancer types and are important regulators of inflammatory responses and mediators of resistance to chemotherapy [88,89,90,91,92,93]. In mammalian cells, IAPs comprise a family of eight members (neuronal apoptosis inhibitory protein (NIAP), cellular inhibitor of apoptosis protein 1 (cIAP1), cellular inhibitor of apoptosis protein 2 (cIAP2), X-linked inhibitor of apoptosis (XIAP), survivin, apollon, melanoma inhibitor of apoptosis protein (ML-IAP) and inhibitor of apoptosis protein-like protein 2 (ILP2)), first identified for their ability to negatively regulate apoptosis [94]. The common feature of all IAPs is the presence of the functional baculovirus IAP repeat (BIR) domains necessary for protein-protein interaction [95]. Five IAPs (cIAP1, cIAP2, XIAP, ML-IAP and ILP2) also possess really interesting new gene (RING)domains with E3 ligase activity enabling them to ubiquitinate protein substrates [96]. XIAP, cIAP1 and cIAP2 are principally involved in anti-apoptotic and pro-inflammatory/pro-survival functions and have been implicated in tumorigenesis and drug resistance [97,98].

XIAP interferes with final steps of apoptosis induction and is the only IAP that inhibits caspase activity by direct binding [75]. XIAP binds to and inhibits effector caspases-3/7 via its linker-BIR2 domain, preventing the final auto-catalytic step of their activation [99]. BIR3 of XIAP interacts with the APAF1/caspase-9 complex to sequester the N terminus of caspase-9 preventing activation of the apoptotic effectors. The cellular IAP proteins (cIAP1/2) can also inhibit caspase activity indirectly through their ubiquitin ligase activity promoting survival signaling. Release of SMAC from the mitochondria following MOMP is required to bind to both the BIR2 and BIR3 domains to release bound caspases and promote cell death execution [75,100,101]. Numerous pre-clinical studies demonstrate that targeting IAPs is an attractive therapeutic approach for several cancer types including colorectal and prostate cancer [102,103]. Various compounds are in development that mimic the natural IAP antagonist, SMAC, to prevent IAP-mediated caspase inhibition and in turn increase apoptotic responses of tumor cells to chemotherapy and, increasingly, immunotherapies. By specifically inhibiting the antiapoptotic activity of IAPs, SMAC mimicking IAP antagonists could offset the protective effect of IAPs [97,100]. IAP antagonists were synthesized as monovalent compounds (LCL-161, Xevinopant (Merck, Darmstadt, Germany), GDC-0917, and GDC-0152) (Genentech, San Franscisco, CA, USA), containing one tetrapeptide moiety (AVPI), or bivalent (Birinapant (Tetralogics Pharmaceuticals, Malvern, PA, USA), HGS-1029 (Human Genome Sciences, Rockville, MD, USA), APG-1387 (Ascentage Pharma, Suzhou, China), containing two tetrapeptide moieties chemically linked [104,105,106,107,108,109,110]. In addition, a small molecule non-peptidomimetic ASTX660 (Astex Pharmaceuticals, Cambridge, UK is progressing clinically.

### 3.1. Monovalent IAP Antagonists

LCL161 (Novartis, Cambridge, MA, USA) was the first orally available IAP antagonist described promoting the degradation of cIAP1 which is now the accepted pharmacodynamic (PD) biomarker for these agents caused by the triggering of cIAP1’s E3 ligase activity to auto-ubiquitinate itself [105,111,112]. The first-in-human trial (NCT01098838) was carried out in patients with advanced solid tumors to assess safety and tolerability. Pre- and post-dose tumor biopsies confirmed rapid and prolonged depletion of cIAP1 for up to 7 days after drug administration. Lack of clinical effectiveness was suggested to be due to a lack of or insensitivity to TNFα, with expression of TNFα predictive of LCL161 sensitivity in vivo [113]. LCL161 moved to Phase II trial in patients with triple-negative breast cancer (TNBC) in combination with paclitaxel. Patients were stratified according to a predictive IAP gene expression signature, with each group randomized to receive neoadjuvant paclitaxel alone or in combination with LCL161. Pathological complete response was achieved in 30% of the positive gene expression signature group; however, significant toxicity, including severe neutropenia, was a concern in the combination arm [114]. A Phase II trial identified that high XIAP expression conferred resistance to LCL161 and that XIAP expression increased in patients that experienced disease progression [76]. IAP antagonists, initially developed to induce apoptosis, have more recently been shown to have broad immunomodulatory effects on both the innate and adaptive immune systems [115,116,117]. IAP antagonists modulate NF-κB activity which can enhance tumor cell killing and the immune status of a tumor by several mechanisms including the conversion of pro-tumoral M2 macrophages to pro-inflammatory M1-like macrophages, signals promoting B-cell survival and the activation of dendritic cells as well as delivering stimulatory signals to T-cells [118]. As such these agents may offer a powerful combination strategy for use with the emerging immunotherapeutic agents, potentially stimulating immune “cold” tumors to be responsive to this class of agents. Currently, LCL161 is being investigated in combination with PDR001, an anti-PD1 agent in non-small cell lung cancer (NSCLC), TNBC and renal cell carcinoma (NCT02890069).

Xevinapant (Debio-1143/AT-406/SM-406) (Merck, Darmstadt, Germany) is a potent, orally available IAP, targeting cIAP1, cIAP2 and XIAP [77,104]. Currently Xevinapant is being evaluated in combination with pembrolizumab (Merck & Co., Kenilworth, NJ, USA) in patients with advanced pancreatic or colorectal cancer (NCT03871959) and in combination with platinum-based chemotherapy and intensity modulated radiotherapy in head and neck cancer (NCT04459715) [119]. Preliminary results from the first-in-human study demonstrated manageable safety in combination with chemotherapy, target occupancy and cIAP1 degradation, and preliminary efficacy; this has led to with the FDA granting breakthrough therapy designation for its use in head and neck cancer in 2020 in combination with cisplatin-based standard-of-care (SOC) treatment [115].

### 3.2. Bivalent IAP Antagonists

Birinapant (TL32711) (Tetralogics Pharmaceuticals, Malvern, PA, USA), a bivalent IAP antagonist, binds to BIR3 of cIAP1 promoting cIAP1 self-ubiquitination and proteasomal degradation [109]. Birinapant demonstrated promising pre-clinical activity in patient-derived xenograft cancer models, with studies also highlighting its synergistic activity with DR receptor agonists and other agents [120,121]. The first in human trial (NCT00993239) of birinapant was conducted in patients with advanced solid tumors or refractory lymphoma. The maximum tolerated dose was determined as 47mg/m^2^, as doses exceeding 63 mg/m^2^ were associated with adverse effects, including headache, vomiting and Bell’s palsy. However, birinapant demonstrated on target activity, showing tumor accumulation and 75% reduction in cIAP1 levels [109]. Subsequent Phase I/II studies have been carried out and, despite demonstrating on-target inhibition in trials, minimal clinical efficacy was observed. These early studies indicate that single agent activity is limited; however there remains potential for combinational treatments with other agents. A current clinical trial is investigating its efficacy in combination with intensity modulated radiation therapy in patients with head and neck cancer (NCT0380774) [122,123].

APG-1387 (Ascentage Pharma, Suzhou, China), a bivalent SMAC mimetic and IAP antagonist blocks the activity of XIAP, cIAP1 and cIAP2 inducing degradation of these proteins and inducing caspase activation. APG-1387 also acts as an immune modulator and, preclinically, has demonstrated synergy in combination with the immune checkpoint inhibitor anti-PD1 [124]. APG-1387 is currently in clinical trials for patients with advanced solid tumors and hematological cancers as a single agent (NCT03386526) and in combination with toripalimab (Shanghai Junshi Bioscience Co., Shanghai, China) in colorectal and NSCLC (NCT04284488) and chemotherapy in patients with advanced pancreatic cancer (NCT04643405).

### 3.3. Non-Peptide Mimetic Small Molecule IAP Inhibitors

Tolinapant (ASTX660) (Astex Pharmaceuticals, Cambridge, UK), an oral non-peptide mimetic small molecule dual IAP antagonist, inhibits the BIR3 domains of both cIAP1 and XIAP with similar potencies [125,126]. Tolinapant induced growth inhibition in several xenograft models of breast cancer and melanoma, with preclinical studies showing a dependence on the presence of TNFα for its effectiveness [78,79]. Assessment of on-target activity was demonstrated by measuring the degradation of cIAP1 in peripheral blood mononuclear cells. Tolinapant showed little single agent efficacy; however, evidence of clinical activity was observed in cutaneous T-cell lymphoma (CTCL) [119,126]. As such, recruitment of patients for a Phase I/II trial is ongoing (NCT02503423) in patients with peripheral T-cell lymphoma (PTCL), CTCL and advanced solid tumors to determine safety, PK, and efficacy. Tolinapant was granted orphan drug designation for the treatment of T-cell lymphomas by the FDA in 2020.

### 3.4. IAP Antagonists—Emerging Resistance and Combination Approaches

Despite promising preclinical data IAP antagonists have had limited clinical success as single agents. Several mechanisms of acquired resistance to IAP antagonists have been described including the upregulation of cIAP2 which initially is degraded along with cIAP1 but can rebound as cIAP1 acts as an E3 Ligase for cIAP2, so in the absence of cIAP1, it is stabilized [98,127] (Table 1). TNF is an essential component for SMAC mediated cell death so tumors with no TNF being inherently resistant to these agents [98]. Success of SMAC mimetics clinically will likely be in combination with other agents. TNFα or TRAIL combinations and TNF-inducing chemotherapies sensitize some resistant cancers to SMAC mimetics [98]. IAP antagonists can also stimulate the non-canonical NF-κB pathway and cause an increase in inflammatory cytokines, such as TNF-α, which as well as mediating cell death can also enhance immune cell recruitment. Several IAP antagonists have demonstrated immunomodulatory effects and offer a potential combination partner for the emerging immune-oncology agents in the clinic. Preclinically, successful combination therapies with chimeric antigen receptor (CAR) T cell therapy has been reported, and clinical trials of IAP antagonists with immune checkpoint inhibitors are ongoing [116]. Combinations of SMAC mimetics with targeted therapeutics such as the BH3 mimetics are also being investigated. Overall, SMAC mimetics have the potential to synergize with several treatments placing them well for continued clinical development.

## 4. Therapeutic Strategies for Promoting Apoptosis Directly—Extrinsic Pathway

### 4.1. Death Receptor Targeted Cancer Therapeutics

Significant efforts to induce extrinsic apoptosis therapeutically have focused on the use of exogenous DR ligands such as recombinant peptide formulations or agonistic antibody approaches targeting specific DRs [128]. Stimulation and subsequent aggregation of DRs by these agents triggers DISC formation and activation of caspase-8 to induce apoptosis. Early studies with tumor necrosis factor (TNF) and agents targeting the Fas (CD-95/Apo-1) death receptor were halted due to dose-limiting toxicities in clinical trials [129,130]. In 1995, the TNF-related apoptosis-inducing ligand (TRAIL/Apo2L) was identified with similarity to other TNF superfamily members and offered an alternative approach to target DRs [80,131]. TRAIL was shown to induce apoptosis of cancer cells specifically with minimal normal cell toxicity, suggesting a significant therapeutic window for this class of agents [132].

Five TRAIL DRs have been documented: Death Receptor 4 (DR4/TRAILR-1), Death Receptor 5 (DR5/TRAILR-2), Decoy Receptor 1 (TRAILR-3/DcR1), Decoy Receptor 2 (TRAILR-4/DcR2) and osteoprotegerin (OPG) [133]. TRAIL belongs to the tumor necrosis superfamily (TNFSF) and can bind to its associated DRs in two forms: membrane-bound or soluble TRAIL, with the membrane-bound form (normally expressed on the surface of immune effector cells such as natural killer and CD8+ T-cells [132]) the more active. Trimerization of TRAIL monomers is crucial to induce TRAIL-R clustering; however, only two of the five TRAIL DRs, DR4 and DR5, can provoke an apoptotic response as they are the only TRAIL receptors with functional cytoplasmic DDs [134,135]. The selective apoptotic activation caused by DR4/DR5 ligand binding on cancer cells renders both DR4 and DR5 as desirable therapeutic targets in cancer therapy. Recombinant TRAIL formations were developed that could bind both DR4 and DR5 and recombinant antibodies designed to target specific receptors.

#### 4.1.1. First Generation TRAIL Agonists

Dulanermin (rhApo2L/TRAIL/AMG-951) (Genentech, San Franscisco, CA, USA), a recombinant soluble TRAIL variant comprising the TNF homology domain within the extracellular region of human TRAIL (amino acids 114–281) is capable of forming stable biologically active trimers and can bind both death receptors, DR4 and DR5, triggering apoptosis offering a broader spectrum of activity than targeting a single DR alone [136,137]. Preclinically, dulanermin demonstrated cancer cell-selective cell death while sparing normal cells and synergized with various chemotherapeutics, demonstrating its potential as a therapeutic [137,138]. The first-in-human trial demonstrated an acceptable safety profile with manageable adverse effects (AE). The half-life was short (0.5–1 h) with no accumulation in serum observed with repeated cycles [139,140,141,142,143]. Disappointingly, all phase II studies concluded that Dulanermin did not add to the antitumoral effects seen with conventional treatments alone [144,145,146]. However, a phase III study carried out by Shanghai Gebaide Biotechnology (Shanghai, China) observed optimistic results with dulanermin as a possible first line treatment option for untreated NSCLC, with improved progression-free survival and objective response rates but not overall survival (OS) when patients were treated with dulanermin, cisplatin and vinorelbine combinations compared to chemotherapy alone. A subsequent phase III trial aims to determine whether dulanermin can improve the OS of previously treated advanced NSCLC patients (NCT03083743) [147]. Overall, although well-tolerated and deemed safe in clinical trials dulanermin demonstrated limited efficacy [136,139,140,141,142]. Its weak agonistic activity, poor PK profile and short half-life was attributed to its inability to form higher order complexes [81,82]. In addition, it can also bind to the non-apoptotic TRAIL receptors DcR1, DcR2, and OPG potentially diluting its binding concentration to DR4 and DR5.

#### 4.1.2. Second Generation rTRAIL Preparations

Early agents demonstrated the safety of TRAIL targeted therapeutics however they were generally clinically ineffective. Several approaches using various N-terminal tags were explored to facilitate the purification of the recombinant TRAIL formulations and improve stability including poly-histidine or FLAG epitope tags or leucine and isoleucine zipper (lz/izTRAIL) tags [148]. Second generation agents while demonstrating superior receptor clustering, enhanced cell death and promising preclinical activity, including improved serum half-life (1.3 h) with no limiting toxicities, have also met significant challenges. Alternative approaches to improve the in vivo properties of TRAIL involved covalently linking TRAIL to molecules with favorable PK properties such as polyethylene glycol (PEG). Pegylated versions of izTRAIL were formulated that further improved the stability and efficacy over the unpegylated version [132,148]. Circularly permuted TRAIL (CPT) consisting of the N-terminus amino acids 121–135 of TRAIL and the C-terminus amino acids 135–281 connected via a flexible linker engages both TRAIL-R1 and TRAIL-R2 as stable homotrimers also demonstrated safety in clinical trials.

#### 4.1.3. DR4/DR5 Antibody Agonists

A range of DR4 or DR5 specific agonistic monoclonal antibodies were developed as an alternative method to target and activate the TRAIL receptor pathway [149,150,151,152,153]. These targeted antibodies have an extended half-life (6–21 days) compared to recombinant TRAIL formulations. Here we discuss a number of DR4/DR5 agonistic antibodies in clinical trials.

Mapatumumab (HGS1012/HGS-ETR1/TRM-1) (Human Human Genome Sciences, Rockville, MD, USA) has been the only fully human DR4 agonistic antibody to progress clinically. Preclinically single-agent treatment of mapatumumab resulted in tumor regression of xenograft models and enhanced the tumor efficacy of conventional chemotherapies [149] however in multiple phase I/II studies although safe, mapatumumab was found to be ineffective as a single or combination agent in a variety of human cancers [154,155,156,157,158,159,160,161,162,163]. For example, a phase II study in NSCLC patients in combination with carboplatin and paclitaxel demonstrated no additional benefit of adding mapatumumab to SOC treatments [160]. Several DR5 agonistic monoclonal antibodies have been developed including tigatuzumab (Daiichi-Sankyo Co. Ltd., Tokyo, Japan), conatumumab (Amgen, Thousand Oaks, CA, USA), LBY135, drozitumab (Genentech, San Franscisco, CA, USA and lexatumumab (Human Genome Sciences, Rockville, MD, USA) with limited clinical success. Conatumumab, developed by Amgen and licensed to Takeda Pharmaceuticals (Nihonbashi, Tokyo, Japan) binds specifically to the extracellular death domain of DR5. Preclinical studies demonstrated efficacy in cancer models enhancing the antitumor activity of several cancer therapeutics including 5-fluorouracil, irinotecan, and gemcitabine. Phase I studies demonstrated its safety in patients as a single agent and in combination with certain chemotherapies; however, clinical development was discontinued in 2011 due to lack of clinical effectiveness [136,164].

#### 4.1.4. Recent Multivalent TRAIL Targeted Therapeutics under Clinical Investigation

ABBV-621 (APG880) (AbbVie, North Chicago, IL, USA), a first-in-class DR agonist, is a fusion protein comprised of a TRAIL receptor agonist, comprising six receptor binding domains, fused to the Fc domain of a human immunoglobulin G1 (IgG1) antibody. ABBV-621 binds both DR4 and DR5 on cancer cells inducing tumor cell-specific apoptosis. Originally developed by Apogenix (Heidelberg, Germany), a licensing agreement was initiated in 2014 for its clinical development with AbbVie (North Chicago, IL, USA) [165,166]. Initial clinical studies demonstrated an acceptable toxicity profile with evidence of antitumor activity and effects on the blood based pharmacodynamic (PD) markers of apoptosis (cleaved cytokeratin products M30/M65) [167]. The first-in-human study evaluated ABBV-621 both as a single agent and in combination with SOC (NCT03082209). Chemotherapy combinations included the 5-fluorouracil, oxaliplatin, irinotecan (FOLFIRI) regime and bevacizumab (Genentech, San Franscisco, CA, USA) in *KRAS* mutant colorectal cancer patients and venetoclax in hematological cancers. Binding of ABBV-621 to decoy receptors on neutrophils was also reported 2 h post dosing followed by dose-dependent desaturation of receptors in patients at 48–168 h. Preliminary antitumor activity was reported in combination with venetoclax in patients with relapsed or refractory AML [168]. The Phase 1 study is ongoing and currently recruiting.

Genmab (Copenhagen, Denmark ) developed a HexaBody-DR5/DR5 compound, a 1:1 mix of two humanized non-competing DR5 specific monoclonal antibodies each carrying E430G mutations, to enhance hexamerization and induce superior receptor clustering. The agent demonstrated activity in a broad range of cancer cell lines and in in vivo xenograft models [83]. A two-part clinical trial to assess the safety of GEN1029 is ongoing (NCT03576131) comprising of a dose escalation part (phase 1, first-in-human (FIH)) and an expansion part (phase 2a) which will be initiated once the recommended phase 2 dose has been determined.

A tetravalent DR5 agonistic antibody, INBRX-109 (Inhibrx, La Jolla, CA, USA), has recently entered clinical development. INBRX-109, an engineered tetravalent single domain antibody-based therapeutic agonist of DR5, can potently antagonize DR5 through receptor super-clustering. A phase 1 trial is currently evaluating the single agent efficacy of INBRX-109 in solid human tumors including colorectal, and pancreatic adenocarcinomas (NCT03715933). In the initial dose escalation phase, the agent was well-tolerated, and no significant liver toxicities observed at doses up to the maximum administered dose of 30 mg/kg. In 2021, INBRX-109 was granted fast track designation status for the treatment of unresectable or metastatic conventional chondrosarcoma with a registration-enabling Phase 2 randomized, blinded, placebo-controlled study due to commence later this year.

Overall, the more recent multivalent agents with super agonistic activity offer promise to deliver a clinically active agent with an acceptable safety profile.

#### 4.1.5. Death Receptor Therapeutics-Emerging Resistance and Combination Approaches

Several challenges impeded the progress of TRAIL targeted therapeutics including the inability of 1st generation agents to trigger effective receptor cross-linking to induce a significant apoptotic response (Table 1). The superior 2nd generation multivalent TRAIL-R agonists which trigger super-clustering of DR-mediated apoptotic DISC formation and efficacy may overcome the clinical resistance observed with the first-generation agents. Enhancing the half-life and stability of third generation agents may further enhance the clinical efficacy of agents. Alternative methods such as nanoparticles (NPs) with death receptor agonists on their surface are being explored by us and others. By clustering the receptor paratopes on their surface, anti-DR5 decorated NPs have been shown to potently induce apoptosis. Moreover, chemotherapeutic agents can be entrapped within the NP further increasing their anti-cancer activity [84,169].

In general, cancer cells express higher levels of TRAIL DRs relative to normal cells, although reports of high levels of TRAIL receptors on hepatocytes, brain cells and keratinocytes [170,171] raised safety concerns early in the development of this class of agents. Despite promising preclinical data, early clinical trials with 2nd generation TRAIL-R agonists demonstrated disappointing efficacy with a number being halted, such as the novel DR5 targeting tetravalent Nanobody^®^ agonist, TAS266 (Ablynx, Ghent, Belgium), due to unexpected but reversible hepatotoxicity. The mechanism of hepatoxicity was speculated to be related to immunogenicity, the high activity of the compound and levels of DR5 expression on hepatocytes [171]. More recent multivalent agonists such as INBRX-109, a tetravalent DR5 agonistic antibody which is engineered to avoid recognition by self-anti-drug antibodies, offer the potential of delivering superior clinically active agents with acceptable safety profiles.

Non-canonical TRAIL dependent functions including the activation of pro-inflammatory, signaling pathways via NFκB, AKT, MAPK and JNK enhance the malignant phenotype of the cancer through increased proliferation, migration, invasion, and metastases [86]. The emergence of alternative functions of TRAIL other than apoptosis may produce undesirable effects in the context of anti-cancer therapeutics. In *KRAS* mutant cancers, endogenous TRAIL and its receptors have been highlighted to promote tumor growth and metastases by activating Rac1 and promoting migration and invasion of cancer cells, with a direct correlation seen between the level of expression of TRAIL-R2 and the extent of metastasis seen in patients [82,86,148,172]. Novel approaches targeting both KRAS signaling and TRAIL to stimulate the immune response have been suggested [86].

Several combination approaches with alternative treatments have been reported to sensitize cancer cells to TRAIL-induced apoptosis mainly by down-regulation of anti-apoptotic proteins and/or up-regulation of the TRAIL receptors [144,173]. Many cancer cells are intrinsically resistant to TRAIL-targeted therapy or acquire resistance following treatment. Intrinsic TRAIL resistance of cancer cells has been attributed to such factors such as high levels of decoy receptors and the presence of anti-apoptotic proteins, such as FLIP, which can inhibit caspase-8 activation at the DISC. FLIP is frequently overexpressed in cancers, often in therapy-resistance settings, and as such, is an attractive anti-apoptotic protein to target therapeutically [174]. Several studies have shown that silencing FLIP by siRNA approaches sensitizes cancer cells to TRAIL and other anti-cancer therapeutics [174,175,176,177]. Several agents are known to downregulate FLIP expression, including certain chemotherapeutics in specific genetic contexts and histone deacetylase inhibitors, and have demonstrated the principle that FLIP down-regulation can be effective and tolerated in preclinical studies. More recently, our group has developed first-in-class small molecule inhibitors capable of disrupting FLIP recruitment to the DISC in cancer cells and inducing caspase-8 and FADD-dependent apoptosis. These FLIP inhibitors can induce apoptosis as a single agent but also promote TRAIL-induced apoptosis highlighting their potential as a combination partner with TRAIL targeted therapeutics [12,13,87,178]. Recently, in genome-wide CRISPR screens, FLIP was identified as the top dependency in *KRAS* mutant versus *KRAS* wild-type cancers [179]. Notably, we have found that these agents also synergize with KRAS G12C inhibitors and 3rd generation EGFR-targeted agents in *KRAS G12C* and *EGFR* mutant NSCLC models [180]. Synergies with standard-of-care chemotherapeutics in colorectal cancer have also been observed, and FLIP was identified as a major resistance mechanism to CAR-T cell therapy [180]. Thus, targeting FLIP has potential beyond the obvious combinations with TRAIL receptor agonists.

## 5. Conclusions

**Figure 4:**
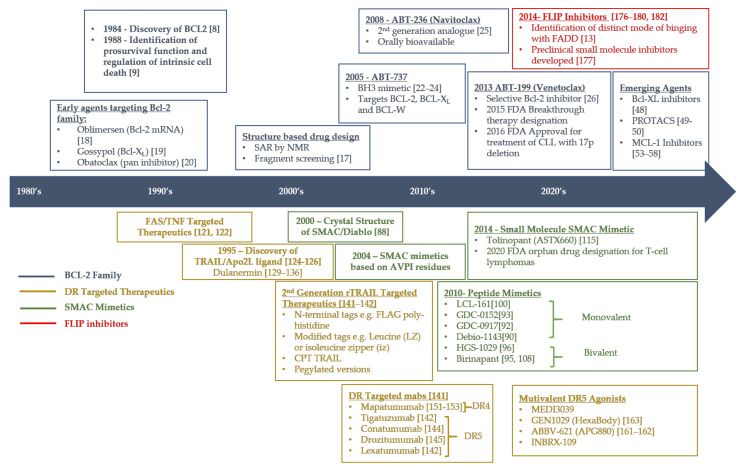
Timeline of progress in therapeutics targeting the apoptotic core machinery. This figure summarizes some of the major advances in the development of different therapeutic agents targeting key anti-apoptotic proteins from the early 1980’s to the current time. Abbreviations:B-cell lymphoma family (BCL-2), BCL-2 associated X protein (BCL-XL), B-cell lymphoma-w (BCL-W), BCL-2 homology domain-3 (BH3), Chronic Lymphocytic leukaemia (CLL), Circularly permuted TRAIL (CPT), Death receptor (DR), US Food and Drug Administration (FDA), Fas-associated death domain (FADD)-like IL1β-converting enzyme-inhibitory protein (FLIP), messenger RNA (mRNA), Second mitochondria-derived activator of caspases (SMAC), Tumor necrosis factor (TNF)-related apoptosis-inducing ligand (TRAIL).

Directly targeting anti-apoptotic proteins like Bcl-2, IAPs and FLIP or pro-apoptotic effectors like DR4/5 are attractive anti-cancer strategies (Figure 4). Drug development targeting inhibitors of the apoptosis pathway has been challenging due to fundamental issues, such as lack of enzymatic activities necessitating protein-protein interaction inhibition and protein degradation approaches, and significant crosstalk between pathways and redundancies within pathway. Crucial to the future success of this class of agents will be the development of reliable pharmacodynamic markers to monitor drug efficacy (such as cIAP1 for IAP antagonists) and target engagement and predictive biomarkers to select patients with tumors likely to be responsive to these agents. As for every targeted agent so far developed, emergence of resistance to this class of agents is inevitable and using rational combination strategies to overcome these mechanisms of resistance will be the key to their clinical success.

## Figures and Tables

**Table 1:** Common acquired resistance mechanisms to anti-apoptotic targeted agents.

Drug Class	Resistance Mechanism	Mechanism to Overcome Resistance	References
BH3 Mimetics	Compensatory anti-apoptotic response Increased Livin (IAP), MCL-1, BCL-X_L_ levels Decreased BCL-2 levels Mutations in regulatory proteins altering their binding site G101V/F104L mutations in BCL-2 binding groove BAX mutations e.g., G179E Distinct mitochondrial metabolic profile Increased anaerobic glycolysis leading to protection against mitochondrial membrane depolarisation Morphological changes e.g., increased cristae	Combinations with other pathway targeted therapeutics Combinations with chemotherapy, radiotherapy, or targeted agents Structure based design of novel agents targeting mutant variants	[17,33,41,58,64,72]
IAP Antagonists	Low TNF levels inherent resistance Compensatory upregulation of other IAP’s Activation of non-canonical NF-κB signalling Increased cIAP2 expression Altered cytokine secretions	Combinations with other agents including pathway relevant therapeutics, chemotherapy, and immune checkpoint inhibitors Sensitisation with TNF or TRAIL therapeutics Inhibition of NF-κB through pharmacological inhibitor of IκB e.g., BMS-345541	[75,76,77,78,79]
Death Receptor Targeted Therapeutics	Changes in death receptor expression levels Epigentic silencing of DR4 Clathrin mediated endocytosis Post-translational modifications Decoy Receptors Upregulation of intracellular anti-apoptotic proteins e.g., FLIP, BCL-2, Other death/survival mechanisms e.g., autophagy	Superior multivalent agonists—increased potency and PK properties Alternative delivery methods e.g., nanoparticles Combination strategies Other targeted therapeutics e.g., EGFR inhibitors, PARP inhibitors Other pathway relevant agents e.g., FLIP inhibitors Chemotherapies/Radiotherapy Autophagy inhibitors	[80,81,82,83,84,85,86,87]

## Abbreviations

AE	Adverse Effects
AML	Acute myeloid leukemia
APAF1	Apoptotic protease activating factor-1
BAK	BCL-2 antagonist killer 1
BAX	BCL-2 associated X protein
BCL-2	B-cell lymphoma family
BCL-W	B-cell lymphoma-w
BCL-X_L_	B-cell lymphoma-extra large
BH	BCL-2 homology domain
BID	BH3 interacting-domain death agonist
BIM	Bcl-2-like protein 11
BIR	Baculoviral IAP repeats
Caspase	Cysteinyl aspartate specific protease
cIAP	Cellular inhibitor of apoptosis
CLL	Chronic lymphocytic leukemia
CPT	Circularly permuted TRAIL
CTLC	Cutaneous T-cell lymphoma
DD	Death domain
DED	Death effector domain
DISC	Death inducing signaling complex
DR	Death receptor
DR4	TRAIL-R1 (Death receptor 4)
DR5	TRAIL-R2 (Death receptor 5)
FADD	Fas-associated death domain
FDA	US Food and Drug Administration
FIH	First-in-human
FLIP	Fas-associated death domain (FADD)-like IL1β-converting enzyme-inhibitory protein
IAP	Inhibitor of apoptosis
IND	Investigational new drug
ILP2	Inhibitor of apoptosis protein-like protein 2
ITD	Internal tandem duplication
izTRAIL	Isoleucine zipper TRAIL
lzTRAIL	leucine zipper TRAIL
MCL-1	Myeloid cell leukemia sequence 1
ML-IAP	Melanoma inhibitor of apoptosis protein
MM	Multiple myeloma
MOMP	Mitochondrial outer membrane permeabilization
NF-κB	Nuclear factor-kappa B
NIAP	Neuronal apoptosis inhibitory protein
NOXA	Phorbol-12-myristate-13-acetate-induced protein 1
NSCLC	Non-small cell lung cancer
Omi	Human serine protease high temperature requirement A (HTRA2)
PBMC	Peripheral blood monocytes
PD-L1	Programmed Death Ligand-1
PEG	Polyethylene glycol
PK	Pharmacokinetic
PTCL	Peripheral T-cell lymphoma
PROTAC	Proteolysis targeting chimera
PUMA	p53 upregulated modulator of apoptosis
RING	Really interesting new gene
SCLC	Small cell lung cancer
SMAC	Second mitochondria-derived activator of caspases
SOC	Standard of care treatment
tBID	Truncated BID
TNBC	Triple negative breast cancer
TNF	Tumor necrosis factor
TNFSF	Tumor necrosis superfamily
TRAIL	Tumor necrosis factor (TNF)-related apoptosis-inducing ligand
Treg	Tumor infiltrating regulatory T cells.
XIAP	X-linked inhibitor of apoptosis
