# Correction: Hamilton et al. Therapeutics Targeting the Core Apoptotic Machinery. *Cancers* 2021, *13*, 2618

**DOI:** 10.3390/cancers14061441

**Published:** 2022-03-11

**Authors:** Claudia Hamilton, Jennifer P. Fox, Daniel B. Longley, Catherine A. Higgins

**Affiliations:** Patrick G. Johnston Centre for Cancer Research, Queens University, Belfast BT9 7BL, UK; chamilton40@qub.ac.uk (C.H.); Jfox14@qub.ac.uk (J.P.F.); d.longley@qub.ac.uk (D.B.L.)

There was an error in the original publication [1]. In the Section 4.1.5 “Death Receptor Therapeutics-Emerging Resistance and Combination Approaches”, Paragraph 2, Inhibrx were incorrectly named as the developer of TAS266. The TAS266 compound was developed by Ablynx NV through a collaboration with Novartis AG, not Inhibrx. The text has been updated to reflect this and an associated reference for this compound has also been updated to the correct reference. An additional sentence has been added to the section to reflect that this did not halt the development of this class of agents and highlight improvements in the more recent compounds in development.

The corrected Paragraph 2 in Section 4.1.5 is listed below:

In general, cancer cells express higher levels of TRAIL DRs relative to normal cells, although reports of high levels of TRAIL receptors on hepatocytes, brain cells and keratinocytes [170,171] raised safety concerns early in the development of this class of agents. Despite promising preclinical data, early clinical trials with 2nd generation TRAIL-R agonists demonstrated disappointing efficacy with a number being halted, such as the novel DR5 targeting tetravalent Nanobody^®^ agonist, TAS266 (Ablynx, Ghent, Belgium), due to unexpected but reversible hepatotoxicity. The mechanism of hepatoxicity was speculated to be related to immunogenicity, the high activity of the compound and levels of DR5 expression on hepatocytes [171]. More recent multivalent agonists such as INBRX-109, a tetravalent DR5 agonistic antibody which is engineered to avoid recognition by self-anti-drug antibodies, offer the potential of delivering superior clinically active agents with acceptable safety profiles.

The authors apologize for any inconvenience caused and state that the scientific conclusions are unaffected. The original publication has also been updated.
